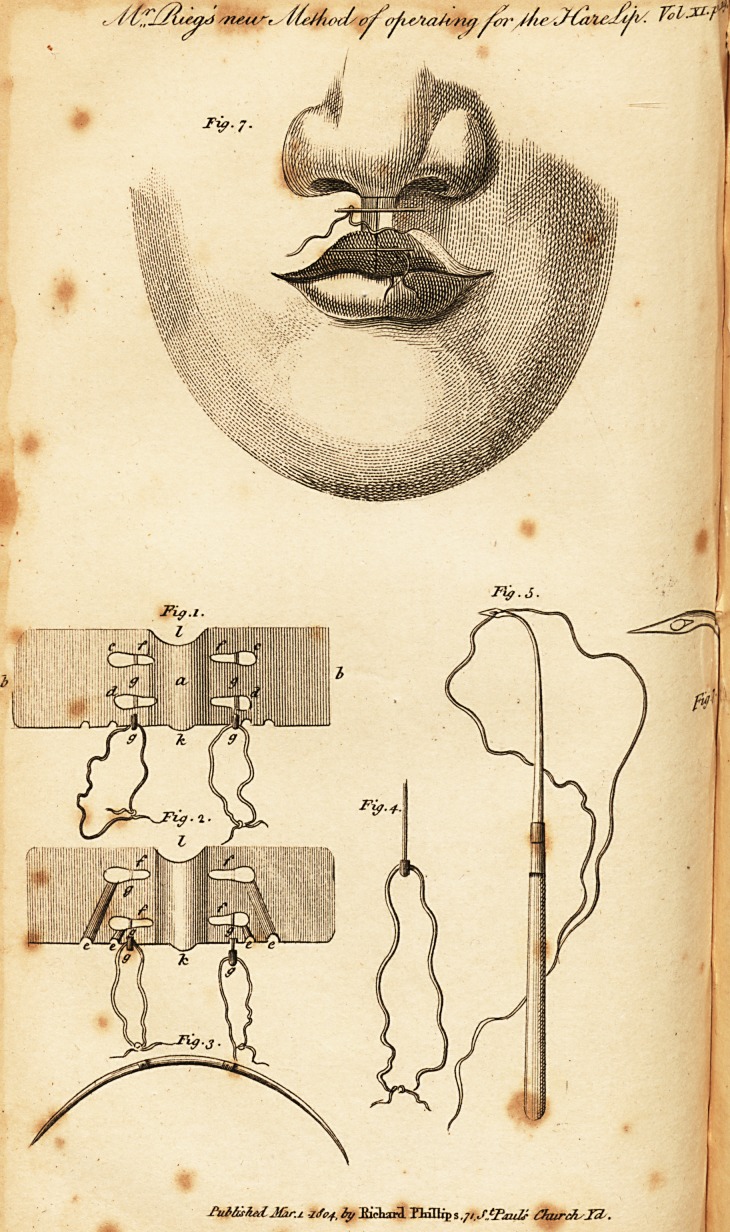# On a New Method of Operating for the Hare-Lip, (Labium Leporinum)

**Published:** 1804-03-01

**Authors:** 

**Affiliations:** Surgeon, at Mentz


### Medical and Phyfical Journal,

VOL. XI.] MARCH 1, 1804. [NO. LXI.

*Printed for R. PHILLIPS, by W. Thorne, Red Lion Court, Fleet Street, London.*

### *On a* NEW METHOD *of* OPERATING *for the* HARE-LIP, *(Labium Leporinum) by* MR. RIEG, *Surgeon, at Mentz.*

[ With an Engraving. ]

AMONGST the different methods proposed for the operation of the hare-lip, the bloody twisted-suture has been approved, as the oldest and most convenient mode of cure, and has received several improvements and alterations in modern times. But this operation requires much accuracy and care, the least mistake being attended with disagreeable consequences; it is also liable to very material objections. In the first place, it seems to be directly opposite to the very purpose of the operation, that we should find no other means to resist the contraction of the adjoining muscles but in the sutures, by which the edges of the wound are intended to be kept in close contact with each other; and thus we endeavour to accomplish in this way two intentions, the former of which must always counteract the scope of the latter; a circumstance very often experienced in the cure of that deficiency. It may be farther alleged, that by the method of cure which has hitherto been in practice, a great deal of irritation and pain is caused, and it has often happened, that even in favourable cases, the cicatrix became uneven and puckered. In order to prevent these inconveniences, which may arise from all the known methods in many instances which we find recorded, I venture to propose a new mode of treatment, by which not only an effectual and certain cure is obtained, but which is likewise attended with less pain and difficulty in the application.

After having separated the adhesions of the upper lip with the gums, and cut off the sides of the fissure, so as to reduce it to the state of a recent wound through the whole extent of it, a small plate of silver, of the same breadth and length with the upper lip, and curved so as to lie close on the arch of the upper jaw, is placed under the upper lip, fig. 1. Both ends, and also the corners of this plate, must be round and blunt, to prevent any harm or inconvenience, which it may otherwise occasion to the patient. On both sides of the plate small holes are made, two of which a little nearer to the superior margin, and the two other nearer to the inferior margin, and the distance between those of one side from those of the other makes one third of the whole length of the plate, or about one inch, *a b c d*. This plate serves to keep the lip in its natural situation, and at the same time to render the application of the adhesive plasters and of the uniting ligatures much easier, by covering any unevenness made by the teeth and gums, but especially to prevent the ligatures from cutting or irritating the parts through which they are passed.

The plate being thus placed under the upper lip, the surgeon must take care to stretch the lip equally on it, that the sides of the fissures exactly correspond with each other, and to bring the edges of the sore close together, which is greatly facilitated by the assistant pushing forward the cheeks. Mean while two waxed threads are provided at each end, with flat, double edged, and sharp needles; or at one end may be applied the needle, fig. 5. The surgeon now takes one side of the lip, together with the subjacent plate, either with the thumb and fore-finger of the left hand, or with Bell's curved forceps, and introduces with his right hand, by slightly lifting up the plate, the point of the needle into a groove which is on the inferior superficies of the plate *e*, till he arrives at the superior hole on that side, through which the needle being pushed, he perforates the upper lip and at the same time a small piece of sponge; the needle being detached from the thread, the latter hangs down the upper lip. This being done, the surgeon takes the needle of the other end of the same thread, and passes it in the same way, through the inferior hole of the same side; and having disengaged the needle from the thread, he gives the two ends of the thread to the assistant.

Before the threads are passed through the other side, the surgeon is to see that it is equally stretched above the plate, and thus drawn and pressed to the other side, that the edges of the wound, situated between both threads, may be easily and closely united. This being performed, the surgeon introduces the threads at the other side, in the same manner as before. In order to promote the accurate contact of the edges of the sore throughout its whole extent, tent, incisions, *f*, are adapted on the plate, running from the holes towards the middle, by means of which the threads, if drawn tight, contribute towards pressing the edges of the wound more closely together. To prevent the thread from pressing or cutting the lip at one side more than at the other, the incisions, *f*, may be shut up by means of pins on each side, *g*, provided at one end with a knob, perforated with a small hole, through which a thread may be passed, in order to draw the pin more easily out, if required.

If by drawing the threads tight, it should be found that the edges of the wounds are not in close contact throughout the whole extent, one of the pins may be drawn out at the side where it may be thought most proper; and if this does not suffice, the pin at the opposite side may likewise be drawn out, by which means the edges of the fissure can be brought on each side one line and a half nearer to each other. Where the thread issues from the lip, a small piece of sponge is placed, serving to diminish the pressure on this part. When the margins of the wounds have been closely and equally united, the surgeon is to tie the undermost threads with a chirurgical knot (while the upper threads are drawn tight), and afterwards the upper ones; the knot however being made at the side of the wound, fig. 7. The union of the edges is besides greatly supported by oblong pieces of linen, spread with good adhesive plaster, and reaching from one ear to the other.

When there is a great deficiency of parts, and when the edges of the wound are brought with difficulty together, the following plaster bandage may be applied with advantage. Two large pieces of leather, narrow at one extremity, and reaching to where the threads issue out of the lip, are spread with good adhesive plaster, and on them compresses of the same size should be fastened; at the narrow end, three threads are fixed, crossing each other on the wound, and are fastened to a double headed bandage, which becoming by degrees broader, ends in two small heads. Having applied the leather on the cheek, this bandage is fastened on the compresses by means of pins, while its smaller heads are carried round the neck and head circularly. This bandage is not so easily loosened; and without much incommoding the patient, it has the advantage of supporting the muscles of the cheek, and preventing their retraction. If the fissure is not in the

O 2 middle middle of the lip, the plate should be purposely made, so as to be adapted to the particular case.

In applying the plate, it is of much importance to have the lips sufficiently stretched and tightly drawn together, when the needle is pushed through the hole; but this must be particularly observed in perforating the other part of the edges of the sore, otherwise they will become uneven. This, however, may be always prevented by proper attention; but with a view of guarding against it as far as possible, a small round prominence, *k*, is adapted to the inferior margin of the plate, serving as a guide how far the edges of the fissure are to be extended. It is unnecessary to draw the margins of the sore together before the needle is carried through, but only to bring the plate under one portion of the lip, till the inferior margin of the fissure covers that prominence, or reaches a little above it; while the ligature is only introduced but not drawn tight. Having in the same manner laid the plate under the other portion of the lip, the two edges of the sore will, on drawing the ligature tight, be closely and accurately united. In the middle of the superior margin of the plate an hemispherical segment is cut out, by which it is intended to give the lip full liberty to adhere to the gums, or to receive in it the frenulum. The plate may be made of gold, silver, or any other convenient metal, and also of whalebone, &c. and can be likewise conveniently used in the case of a double hare-lip.

The advantages which seem to arise from this mode of operation are the following: 1. The edges of the wounds are kept in close contact with each other, without being irritated by the retraction of the adjoining muscles. 2. The manner in which the needle is passed through, gives the least pain. 3. The irritation being less, not so great a degree of inflammation occurs as is caused by the common method. 4. The troublesome and sometimes dangerous oozing of the blood is more easily prevented by this than by the common method; and also the sucking of the blood, which is, in some cases, attended with dangerous consequences. 5. The plate prevents the new and disagreeable adhesions of the lip to the gums, which has sometimes taken place after the operation. 6. The bandage is not so readily loosened; and much trouble is saved, as it is not necessary to have needles, the points of which must be either removed or sufficiently covered, in order to prevent their wounding the contiguous parts. With respect to the subsequent treatment and the removal of the ligatures, nothing new or particular is required.

EXPLA-

### EXPLANATION OF THE PLATE.

Fig. 1. The curved plate, made of silver, &c; *a*, the middle part, making about one-third of its whole length; *b*, its margin, which must be made thinner than the middle part; *c*, the two superior holes; *d*, the two inferior.

Fig. 2. Shows the concave inferior superficies of the plate; *e*, the grooves which lead to the holes; *f f f f*, the conical incisions, each being about one line and a half long; *g g g g*, the pins for shutting the incisions; *k*, the round process; *l*, the hemispherical excavation.

Fig. 3. Shows the thickness and curvature of the needle.

Fig. 4. A pin drawn out.

Fig. 5. A curved needle, with a flat handle to it.

Fig. 6. The same magnified, showing that it is screwed to the handle.

Fig. 7. The appearance of the parts, after the ends of the ligatures are made tight, and the union of the edges of the sore accomplished.

## Figures and Tables

**Fig. 1. Fig. 2. Fig. 3. Fig. 4. Fig. 5. Fig. 6. Fig. 7. f1:**